# Endothelial Cell Mechano-Metabolomic Coupling to Disease States in the Lung Microvasculature

**DOI:** 10.3389/fbioe.2019.00172

**Published:** 2019-07-19

**Authors:** David Wu, Konstantin Birukov

**Affiliations:** ^1^Section of Pulmonary and Critical Care, Department of Medicine, University of Chicago, Chicago, IL, United States; ^2^Department of Anesthesia, University of Maryland, Baltimore, MD, United States

**Keywords:** metabolism, pulmonary, endothelial (dys)function, microvasculature, endothelial mesenchymal transition

## Abstract

Lungs are the most vascular part of humans, accepting the totality of cardiac output in a volume much smaller than the body itself. Due to this cardiac output, the lung microvasculature is subject to mechanical forces including shear stress and cyclic stretch that vary with the cardiac and breathing cycle. Vessels are surrounded by extracellular matrix which dictates the stiffness which endothelial cells also sense and respond to. Shear stress, stiffness, and cyclic stretch are known to influence endothelial cell state. At high shear stress, endothelial cells exhibit cell quiescence marked by low inflammatory markers and high nitric oxide synthesis, whereas at low shear stress, endothelial cells are thought to “activate” into a pro-inflammatory state and have low nitric oxide. Shear stress' profound effect on vascular phenotype is most apparent in the arterial vasculature and in the pathophysiology of vascular inflammation. To conduct the flow of blood from the right heart, the lung microvasculature must be rigid yet compliant. It turns out that excessive substrate rigidity or stiffness is important in the development of pulmonary hypertension and chronic fibrosing lung diseases via excessive cell proliferation or the endothelial-mesenchymal transition. Recently, a new body of literature has evolved that couples mechanical sensing to endothelial phenotypic changes through metabolic signaling in clinically relevant contexts such as pulmonary hypertension, lung injury syndromes, as well as fibrosis, which is the focus of this review. Stretch, like flow, has profound effect on endothelial phenotype; metabolism studies due to stretch are in their infancy.

## Introduction

In 2010, cardiovascular diseases accounted for 32% of all deaths (~780,000) in the U.S (NHLBI, 2013). Macrovascular arterial disease pre-disposes to microvascular dysfunction and is classically recognized as a complication in atherosclerosis (Shah et al., 2018), diabetes (Shi and Vanhoutte, 2017), and rheumatoid arthritis (Foster et al., 2010). Not surprisingly, microvascular dysfunction involves dysfunction of the organ proper. The lung has a prominent place in the microvasculature, as its estimated capillary surface area (as defined by diameter of a vessel 10 μm or less) is roughly 50–70 m^2^, which is one-fourth the size of a tennis court (Weibel, 1973). This is 20-times the surface area of all other vessels in the body combined (Albertine, 2016).

Endothelial cells (ECs) line the vasculature, including arteries, veins, and lymphatics. ECs are continuously subjected to shear stress (frictional force per unit area parallel to the cell) which can range from 10 to 50 dyne/cm^2^ in large arteries, 5 to 20 dyne/cm^2^ in the microvasculature, and about 10-fold less in veins (Paszkowiak and Dardik, 2003) [this is variable depending on tissue bed (Remuzzi et al., 1992)]. Shear forces play an especially important role in promoting cell quiescence or activation (Davies, 1995; Chiu and Chien, 2011; Davies et al., 2013). Lymph flow is slower even than microvascular flow, averaging 0.64 dyne/cm^2^ with peaks of 4–12 dyne/cm^2^ (Dixon et al., 2006). The specific aspects of flow are complex, vectorial, varying in both space and time (Noris et al., 1995; Blackman et al., 2000; Feaver et al., 2013; Wang et al., 2013). Furthermore, ECs are transcriptionally specialized according to their organ location (He et al., 2018) and accordingly have different responses to shear stress (Reinitz et al., 2015). ECs in the lungs are subject not only to shear stress due to the continuous pulsations from the cardiac cycle, but also to copious stretch due to the respiratory cycle. Stretch forces on the endothelium play a critical role in lung injury during mechanical ventilation (Acute Respiratory Distress Syndrome Network et al., 2000; Birukov et al., 2003; Slutsky and Ranieri, 2013).

Besides flow and stretch, endothelial cells are also responsive to stiffness (i.e., resistance to deformation) in the underlying extracellular matrix. Compared to hemodynamical forces, stiffness forces are much higher. Stresses and stiffness have the same units of measure, which allows for direct physical comparison. To put this into tactile perspective, 100 kPa is about the stiffness of muscle, whereas 1 kPa is about the stiffness of lung tissue (1 Pa = 0.1 dyne/cm^2^). Thus, stiffness forces are orders of magnitude larger than shear stress. Cellular traction forces or tensile stresses (also force per unit area, but this time a force that tends to lengthen or compress a material) are also much higher than hemodynamic shear stress—typically ~5 kPa at a focal adhesion (Balaban et al., 2001) and endothelial cells exert about 100–300 kPa (Shiu et al., 2004). Intuitively, cellular traction stress should match or be correlated substrate stiffness through force sensing at the focal adhesion (Califano and Reinhart-King, 2010); larger tensile stresses are necessary to overcome substrate stiffness (Balaban et al., 2001). Correspondingly, hemodynamic shear sensor sensors are thought to be activated with much less force (Fang et al., 2019). Substrate stiffness is important in chronic fibrosing lung diseases as well as promoting the endothelial to mesenchymal transformation.

Thus, ECs are mechanosensing machines (Davies, 1995), with the specific mechanical environment having a profound effect on EC phenotype in health and disease. There is now increasing evidence that mechanical forces causing endothelial activation—the conversion of quiescent phenotype to activated phenotype—and endothelial transformation—are associated with a change in cell metabolism (Sawada et al., 2014; Eelen et al., 2015). This review will focus on new developments in shear stress regulation of endothelial metabolism and signaling, and how substrate stiffness affects endothelial cell phenotype. Dysfunction in these pathways leads to the pathogenesis of major diseases of the pulmonary vasculature, including pulmonary hypertension, acute lung injury, and pulmonary fibrosis.

### General Aspects of Endothelial Metabolism

Glycolysis is the pathway that converts glucose to pyruvate and then lactate. ECs are highly glycolytic. This was determined either by directly measuring glucose flux in cultured cells (De Bock et al., 2013b) or by inhibition of various energy pathways and measuring the resulting heat flux via calorimetry. In the latter experiment, it was discovered that inhibiting hexokinase (the first step of glycolysis which traps glucose inside the cell) with 2-deoxyglucose significantly reduced heat generated from ECs (Schrimpf et al., 1994). Furthermore, the glycolysis index (lactate/glucose ratio) in cultured human umbilical vein endothelial cells (HUVECs) is around 1.74 (Kim et al., 2017), suggesting that ECs metabolize glucose almost entirely into lactate. Thus, only a small fraction of glycolytic intermediate pyruvate is metabolized by mitochondria. ECs, in contrary to other cell types, are as glycolytic as tumor cells (De Bock et al., 2013b), and use very little oxygen to generate ATP (Schrimpf et al., 1994; Quintero et al., 2006; De Bock et al., 2013b). In support of this, older electron microscopy studies suggested that the total mitochondrial fraction of ECs is only 4–5% (slightly higher, up to 10% in blood brain barrier ECs) compared to 32% in cardiomyocytes (Oldendorf et al., 1977; Barth et al., 1992). This poses an interesting teleological conundrum as the vasculature holds the highest oxygen tension. One thought is that this evolutionary feature lets oxygen diffuse into the organ tissue instead of being consumed by the endothelium. Another hypothesis is that glycolysis powers the EC migratory or endocytotic machinery, whereas the tricarboxylic acid cycle/oxidative phosphorylation (TCA/OXPHOS) system is used for cell growth and division. In this scheme, glycolytic energy is necessary to continually modulate cell boundaries and perform transcytosis of macromolecules, whereas cell proliferation does not regularly occur in adult vasculature (Kim et al., 2017).

A caveat is that these are all *in vitro* studies and the true *in vivo* glycolytic rate of ECs is unknown. Furthermore, these studies were all done without hemodynamic shear stress and on cell culture plastic, which is 5 orders of magnitude stiffer than muscle. One *in vitro* study looked at blood brain barrier endothelial cells and lactate production and found that shear stress reduced lactate production, indicating that EC metabolism is dynamically modulated by hemodynamic forces and that measuring the metabolic properties of ECs without flow would introduce artifact (Cucullo et al., 2011). Another study found that shear stress increased the fraction of mitochondrial ATP production on ECs *in vitro* (Wu et al., 2017). Furthermore, the natural concentration of lactate in circulating human blood is < 2 mM, with glucose around 5 mM, suggesting a far lower glycolysis index *in vivo* than what is measured *in vitro*.

EC metabolism is a topic of intense, recent investigation (Theodorou and Boon, 2018). Briefly, glycolysis is important in mouse models of angiogenesis and lymphogenesis (De Bock et al., 2013b; Yu et al., 2017). Derivatives of the glycolytic pathway are involved in nucleic acid synthesis, NAD/NADH balance, and O-linked glycolsylation (Theodorou and Boon, 2018). Glycolysis plays a central role in energy and intermediate metabolite generation and is required for activation of a diverse set of cell types including immune cell activation, and tumor cell proliferation and survival (Ghesquière et al., 2014). Mitochondrial metabolism is, broadly speaking, important for cell growth. Amino acid metabolism through the TCA cycle (glutamine and its derivative, α-ketoglutarate) enhance anaplerosis (replenishing of TCA cycle intermediates that have been consumed for biosynthesis), critical for cell proliferation (Huang et al., 2017a; Kim et al., 2017). The electron transport chain (ETC) itself is also required for EC proliferation and angiogenesis as well as redox balance *in vivo* (Diebold et al., 2019). Interestingly, the ETC may be dispensable for EC migration (Diebold et al., 2019). Fatty acid metabolism is critical for *de novo* nucleotide synthesis, which is needed for EC proliferation *in vivo* (Schoors et al., 2015) as well as for generation of acetyl-CoA derivatives for histone acetylation in lymphatic ECs for differentiation and fate maintenance (Xiong et al., 2018).

### Endothelial Cell Phenotype Is Dictated by Flow

In general, flow can be divided into unidirectional flow (high shear stress) and disturbed flow (low shear stress), or atheroprotective and atheroprone (against atherosclerosis), respectively, in the systemic arterial tree (Dai et al., 2004). Cardiovascular flow type has been shown to be critical for angiogenesis (Galie et al., 2014), cardiovascular development (Vermot et al., 2009; Chen et al., 2012; Franco et al., 2015) in zebrafish and mouse models, and plays a central role in the activation and subsequent inflammation of ECs in the pathogenesis of cardiovascular disease both *in vitro* and *in vivo* (Gimbrone and García-Cardeña, 2016). Besides macrovascular disease, flow patterns are also altered in the microvasculature in humans during sepsis (Vincent, 2001; De Backer et al., 2002) or in murine models of acute lung injury (Schmidt et al., 2012).

Tangential shear stress due to flowing blood is proportional to the change in velocity divided by the distance from the vessel wall. High shear stress promotes a healthy endothelium, characterized by quiescence and maintenance of vascular barrier integrity. In contrast, areas of atheroprone or disturbed flow (low shear stress) are sites where the endothelium undergoes “activation,” characterized by inflammation and a reduction in vascular barrier integrity (Dai et al., 2004; Davies et al., 2013). Endothelial cells have a “Goldilocks” set point of preferred shear stress: brain ECs lose barrier function under shear flow when it is twice as much as normal (40 dyne/cm^2^) (Garcia-Polite et al., 2017) and undergo apoptosis under low shear stress in developing zebrafish brains (Chen et al., 2012).

Shear-based endothelial dysfunction-activation (Liao, 2013) in the macrovasculature provides a useful model upon which to extrapolate mechanotransduction biology in microvascular endothelial cells. In the classical shear stress dependence of endothelial pathophysiology, atherosclerosis occurs at sites of disturbed flow: arterial curvature, branching, and bifurcation, characterized by flow separation, transient flow reversals, and low average shear forces (Davies, 1995). Microscopically, ECs align in the direction of flow, both *in vivo* and *in vitro*. *In vitro*, cells align in unidirectional flow producing a highly elongated shape, but not in disturbed flow, resulting in a cobblestone shape (Davies et al., 1986). Interestingly, *in vitro* flow that is perpendicular to the long axis of the cell and long axis of the cytoskeletal elements strongly activates inflammatory pathways (Wang et al., 2013). Failure to align with flow *in vivo* is a hallmark of an atherosclerosis-prone region (Davies, 2008). These atherosclerosis-prone regions have signs of chronic inflammation with increased expression of adhesion molecules and reduction of barrier integrity, all of which develop before any visible signs of disease—thus shear stress by itself could initiate clinical disease (Hajra et al., 2000; Won et al., 2007).

The lung microvasculature flow rate likely has a profound effect on pathophysiology as well. Microvascular endothelial cells are known to migrate against flow at low shear stress toward regions of high shear stress (Ostrowski et al., 2014). Microvascular ECs align with the flow field at lower shear stress levels and, interestingly, perpendicular to shear stress at higher values (>34 dyn/cm2) (Ostrowski et al., 2014). In one study, shear stress and microvascular barrier were fairly independent, but flow opposite of conditioned flow state resulted in a large increase in permeability (Adamson et al., 2013), which is similar to the arterial case described above. Cessation of flow in murine-derived microvascular ECs resulted in production of reactive oxygen species (ROS) and increased proliferation as well as cobblestone morphology (Milovanova et al., 2006). These data together highlight the sensitivity of lung microvascular ECs to shear stress.

### Shear Stress Modulation of Endothelial Metabolism

**Figure 1:**
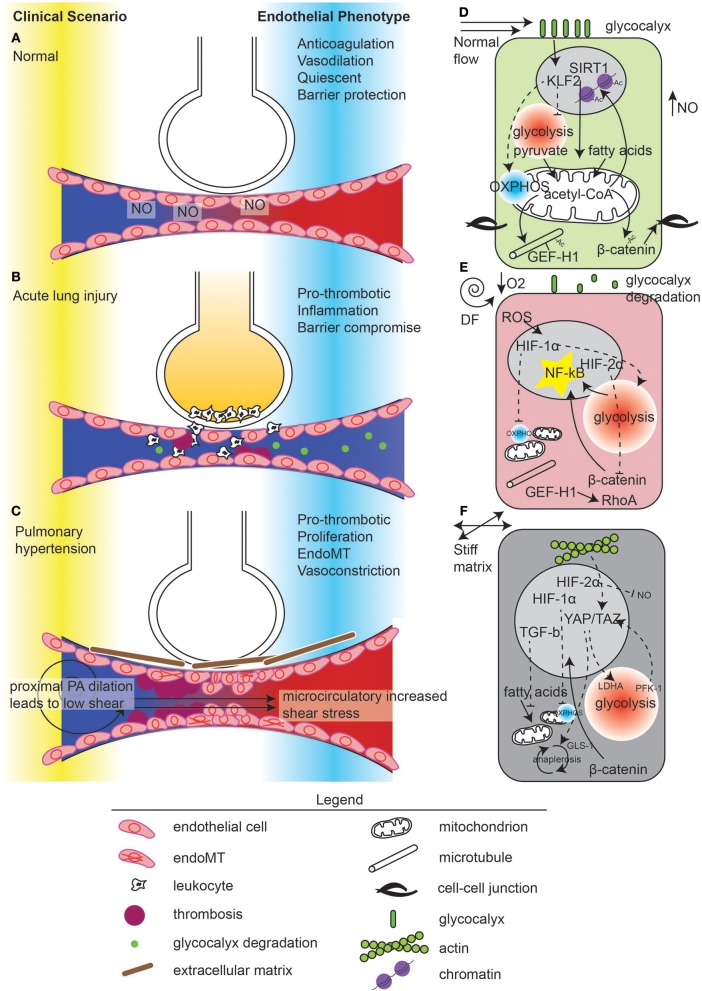
Mechanometabolomic coupling to disease states in the lung.Vascular disease states in the lung in **(A)** normal, **(B)** acute lung injury, and **(C)** pulmonary hypertension. In normal states, ventilation through the alveoli and perfusion occur through nitric oxide (NO) release by the endothelium. Normal microvascular perfusion **(D)** induces a KLF2 high state which suppresses glycolysis and promotes mitochondrial function, leading to pyruvate-derived acetyl-CoA and acetylation of microtubules, β-catenin, and maintenance of quiescence through chromatin modification. In contrast, during acute lung injury **(B)**, flow becomes disturbed due to microvascular dysfunction, manifest by thrombosis and endothelial contraction leading to edema. Edema in the alveolus causes local hypoxia **(E)**. Inflammation leads to glycocalyx degradation, ROS generation which, with hypoxia, activates HIF-1α. HIF-1α suppresses mitochondrial energy generation and induces glycolysis. De-acetylated microtubules release GEF-H1 for RhoA activation. Glycolysis induces NF-κB activation which can also be enhanced by β-catenin nuclear translocation, due to de-acetylation. However, hypoxia also induces HIF-2α, which inhibits β-catenin translocation. In pulmonary hypertension **(C)**, the upstream pulmonary artery is dilated, reducing shear stress. In the microvasculature, flow is thought to be supra-normal due to thrombosis and vessel stiffness, causing endothelial damage. ECs undergo endoMT and synthesize connective tissue, which exacerbates these hemodynamical phenomena. **(F)** ECs in response to increased extracellular matrix stiffness stimulate YAP/TAZ activity which enhances glycolysis and anaplerosis, leading to cell proliferation and endoMT. Hypoxia pathways are also activated in pulmonary hypertension.

The major regulator of unidirectional flow responses of microvascular ECs was discovered in the lung and is named Krüppel-like factor 2 (KLF2) (Dekker et al., 2002; Lee et al., 2006; Parmar et al., 2006). KLF2 or KLF4 [probably functionally redundant in mouse knockouts (Sangwung et al., 2017)] are thought of as master transcriptional regulators that mediate the vasodilatory, anti-inflammatory, and antithrombotic properties of quiescent endothelium (Atkins and Jain, 2007). Reduced expression of KLF2 or KLF4 has been mechanistically linked to increased expression of inflammatory gene markers in both cultured endothelial cells and *in vivo* in both the macro- and microvasculature (Lin et al., 2005, 2010; Parmar et al., 2006; Shen et al., 2009; Villarreal et al., 2010) (Figures 1A,D).

Shear induced metabolic changes to ECs have been studied in the systemic arterial tree, but these lessons may apply to the microvasculature. Generally speaking, metabolic changes in endothelial cells under disturbed flow *in vitro* and *in vivo* include reduced mitochondria mass and function (Chen et al., 2010; Kizhakekuttu et al., 2012), an upregulation of glycolysis via dependence on transcription factor hypoxia inducible factor-1α (HIF-1α) (Feng et al., 2017; Wu et al., 2017), an increase in ROS and advanced glycation end-products (such as in diabetes), and nitric oxide deficiency (Eelen et al., 2015) (such as in atherosclerosis). Additionally, changes in cell metabolism (specifically, upregulation of glycolysis by HIF-1α) are required for endothelial activation and inflammation (Feng et al., 2017; Wu et al., 2017), in addition to angiogenesis (De Bock et al., 2013a,b). Disturbed flow-upregulation of NA(P)DH oxidase 4 (NOX4) and consequent increase in ROS prevents HIF-1α degradation, leading to an increase in glycolytic gene expression. Furthermore, blocking glycolysis by inhibition of major glucose transporter SLC2A1 (GLUT1) can reduce Nuclear Factor-κB (NF-κB) activation in disturbed flow EC models *in vitro* (Wu et al., 2017). Endothelial specific heterozygous HIF-1α mouse demonstrated reduced inflammation and atherosclerosis (Feng et al., 2017). Additionally, HIF-1α, glycolytic gene expression, and inflammatory gene expression are all increased in the regions of disturbed flow in large arteries of swine (Wu et al., 2017).

On the contrary, unidirectional flow has been shown to reduce glycolysis in a KLF2-dependent manner (Doddaballapur et al., 2015) and increased mitochondria biogenesis in rodent endothelium (Chen et al., 2010; Kim et al., 2014). Suppression of glycolysis in activated ECs and ECs exposed to disturbed flow suppresses inflammatory and angiogenic phenotypes (Doddaballapur et al., 2015; Feng et al., 2017; Wu et al., 2017) whereas chemical suppression of ETC function under unidirectional flow increases EC inflammation in cultured flow models (Wu et al., 2017). These data suggest a role for mitochondrial function in modulating endothelial quiescence. However, no analogous genetic experiment has been performed. KLF2 also has a role in shear stress mediated changes in EC metabolism. KLF2 upregulation via disturbed flow represses 6-phosphofructo-2-kinase/fructose-2,6-biphosphatase 3 (PFKFB3), an allosteric regulator of phosphofructokinase 1 (PFK1), which is a critical enzyme in glycolysis (Doddaballapur et al., 2015). PFKFB3 upregulation was shown to promote retinal angiogenesis (De Bock et al., 2013b). Furthermore, blocking PFKFB3 can improve barrier function and reduce cancer adhesion to ECs (Cantelmo et al., 2016). Interestingly, HIF-1α and KLF2 are counter-regulated: KLF2 has been shown to disrupt binding between HIF-1α and its chaperone, HSP90 (Kawanami et al., 2009; Wu et al., 2017).

Due to the relative paucity of mitochondria in ECs, it has been suggested that mitochondria play the role of signaling organelles (Quintero et al., 2006); accordingly, their role in EC biology is complex [angiogenesis, autophagy, ROS signaling, NO bioavailability (Kluge et al., 2013)]. Arterial segments exposed to high shear stress demonstrate increased mitochondrial activity *in vivo* (Kim et al., 2014). Unidirectional flow, in addition to increasing oxidative phosphorylation (OXPHOS), also upregulates mitochondrial biogenesis in a NAD-deacetylase sirtuin 1 (SIRT1)-dependent manner (Chen et al., 2010; Kim et al., 2015). SIRT1 is a key mediator of angiogenesis (Das et al., 2018) and stimulates endothelial NOS activity (Mattagajasingh et al., 2007). Unidirectional flow dynamically increases mitochondrial spare respiratory potential (Wu et al., 2017) probably in a KLF2/4-dependent manner, as KLFs are upregulated under unidirectional flow (Dekker et al., 2002) and increase mitochondrial biogenesis and metabolic function through transcriptional regulation of PPARγ/PGC1 (Liao et al., 2015). Unidirectional flow may also increase ETC activity through HIF-1α degradation, as HIF-1α is known to regulate the ETC by reducing complex I activity (Tello et al., 2011) and by shunting glucose substrates into glycolysis rather than mitochondria. HIF-1α induces pyruvate dehydrogenase kinase 1 (PDK1), which inhibits pyruvate dehydrogenase (PDH), the enzyme that traps pyruvate inside mitochondria as acetyl-CoA (Kim et al., 2006). Interestingly, the activation of SIRT1 is dependent on the cytoskeleton. In response to shear stress, AMPK phosphorylation of cortactin is followed by SIRT1 deacetylation. Due to deacetylation of cortactin, eNOS is able to translocate away from lipid raft domains, increasing NO bioavailability (Shentu et al., 2016).

Further changes in EC metabolism as a response to low shear stress or disturbed flow include an upregulation of transcription factors YAP/TAZ both *in vitro* and *in vivo* (Wang K. C et al., 2016; Wang L. et al., 2016), which also promotes glycolytic metabolism in turn. Yes-associated protein (YAP) and transcriptional coactivator with PDZ-binding motif (TAZ), are modulated by mechanotransduction signaling through the cytoskeleton (Dupont et al., 2011), and promotes a pro-inflammatory EC phenotype (Wang K. C et al., 2016; Wang L. et al., 2016). Nutrient and energy sensing also modulate YAP activity (more below) (Enzo et al., 2015; Wang et al., 2015; Bertero et al., 2016; Santinon et al., 2016). Cancer studies indicate that YAP and HIF-1α may interact to promote glycolysis as YAP may localize to the nucleus and prevent HIF-1α degradation (Zhang et al., 2018b). Interestingly, in the absence of disturbed flow, lymphatic endothelial cells undergo TAZ-dependent proliferation (Sabine et al., 2015).

EC quiescence seems to be associated with a mitochondrial phenotype whereas activation is associated with a glycolytic phenotype. Shear stress modulates the metabolic profile based on the endothelial cell bed. These macrovascular lessons are important for microvascular dysfunction, as macrovascular dysfunction causes disturbed flow in microvascular beds (Siasos et al., 2018). Furthermore, microvascular flow is probably highly altered in acute inflammatory disease processes (Vincent, 2001; De Backer et al., 2002; Schmidt et al., 2012). In animal models of sepsis or acute lung injury [sepsis is the top cause of acute lung injury and has a poor prognosis (Force et al., 2012; Bellani et al., 2016)], lung microvascular flow becomes disturbed or oscillatory (De Backer et al., 2002) and leads to microvascular mitochondrial dysfunction (Arulkumaran et al., 2016). In support of this, lung disease often involves pressure changes in the pulmonary vasculature due to either chronic hypoxic vasoconstriction, chronic thromboembolism, vascular obliteration due to plexiform lesions, or vascular apoptosis. The beneficial metabolic effects of constant high shear stress perfusion may actively repress ischemia sensing pathways and prevent reperfusion injury, perhaps accounting for the success of *ex vivo* lung perfusion technology (Cypel et al., 2011).

Though shear flow, mitochondrial function, and metabolism have been studied in atherosclerosis models of vascular dysfunction, microvascular flow, and metabolism in disease models such as diabetes and pulmonary hypertension or vascular injury are under investigated although glycolytic metabolism in particular is important in ECs in pulmonary hypertension (Xu et al., 2007; Michelakis et al., 2017; Plecitá-Hlavatá et al., 2017; Culley and Chan, 2018).

#### Shear Stress, Endothelial Metabolism, and Acute Lung Injury

Endothelial injury is critical for the promulgation of the acute respiratory distress syndrome (ARDS) during lung injury. One key mechanism is activation of cytoskeletal contractions which disassembles cell-cell junctions and causes ECs to shrink, leading to pulmonary edema (Kása et al., 2015) (Figures 1B,E).

In inflammatory states, the microvascular endothelium is exposed to disturbed flow and KLF2 is downregulated. In sepsis states, there is disseminated EC dysfunction manifested in part by oscillatory flow (Vincent, 2001; De Backer et al., 2002). In an experimental acute lung injury model [intratracheal lipopolysaccharide (LPS)], microvascular dysfunction due to downregulation of KLF2 leads to disruption of barrier function, and lung parenchymal edema formation. Rescue of depleted pulmonary microvascular KLF2, by intravascular delivery of KLF2 mRNA using nanoparticles was able to protect against pulmonary edema and neutrophil infiltration (Huang et al., 2017b). Mechanistically, lung microvascular ECs, when treated with TNFα, are unable to sense shear stress and lose their shear-imposed KLF2 upregulation, leading to loss of barrier function *in vitro* (Huang et al., 2017b).

One possible reason for ECs to becoming insensate to high shear stress is that under TNFα treatment *in vivo*, the pulmonary endothelial glycocalyx is lost (Schmidt et al., 2012). Glycocalyx is an essential part of the flow sensing apparatus in endothelial cells (Florian et al., 2003; Mochizuki et al., 2003; Baeyens et al., 2014; Yen et al., 2015). Endothelial glycocalyx is critical in maintaining capillary fluidity and perfusion homogeneity in the microvasculature (Mcclatchey et al., 2016). Glycocalyx is reduced in sepsis, diabetes, heart failure and sickle cell disease, suggesting a connection between mechanical sensing, NO production, and microvascular perfusion (Cabrales et al., 2007; Mcclatchey et al., 2016). Interestingly, normal shear stress remodeling of the actin cytoskeleton was changed after disruption of the glycocalyx (Thi et al., 2004) and orientation of the cells in response to flow was changed (Moon et al., 2005). Components of the glycocalyx interact with focal adheions (Zimmermann and David, 1999; Rapraeger, 2000) and also with G-protein coupled receptors (Davies et al., 1995). Pulmonary derived glycocalyx shedding may also be systemically toxic and damage other microvascular beds, causing acute kidney injury in clinical studies (Schmidt et al., 2016).

The connection between downregulation of KLF2 in endothelial dysfunction, an increase in HIF-1α-mediated glycolysis and worsened lung injury seems likely however has not been directly shown (Doddaballapur et al., 2015; Feng et al., 2017; Wu et al., 2017). Clinical data indicating that poor gas exchange occurs during acute lung injury suggests that the lung is locally hypoxic (Force et al., 2012). In ARDS, pulmonary lactate is increased, suggesting an upregulation of glycolysis has occurred (Brown et al., 1996; De Backer et al., 1997). Additionally, in a rat proteomic study of acute lung injury, glycolytic enzymes were discovered to be upregulated (Liu et al., 2014). Whereas resting humans have been shown to mount a lactate response to inspired O2 levels around 10% (Huckabee, 1958), HIF-1α stabilization *in vitro* occurs at <5% O2 in hypoxia chambers (Bracken et al., 2006); it is therefore reasonable to assume that tissue hypoxia occurs *in vivo* as tissue oxygenation must be below that of arterial oxygenation. In addition to frank hypoxia, HIF-1α can be stabilized in pseudo-hypoxic states *in vivo* which does not require such extreme levels of hypoxia. This accounts for the findings of focal HIF-1α expression in large arteries of pigs (Wu et al., 2017). Thus, persistent lung (real or pseudo-) hypoxia may allow for local HIF-1α expression (Mcclendon et al., 2017). A role for HIF-1α in the lung was also shown in a septic lymph model of sepsis, also showing that HIF-1α is activated in a pseudo-hypoxic manner (Sun et al., 2017). Compared with control, microvascular ECs treated with shRNA against HIF-1α incubated with septic lymph demonstrated lower levels of TNF-α, IL-6, and IL-1β cytokines, along with increased viability (Sun et al., 2017). Interestingly, HIF-1α in alveolar epithelial cells may be protective against lung injury (Mcclendon et al., 2017; Suresh et al., 2018).

In addition to HIF-1α, HIF-2α is also expressed in lung injury, and may act to protect against vascular barrier dysfunction (Gong et al., 2015). Notably, HIF-2α is also activated by disturbed flow (Wu et al., 2017). HIF-2α upregulation was shown to be protective in a lung injury mouse model as it promotes endothelial barrier maintenance through regulation of a vascular phosphotyrosine phosphatase (VE-PTP or PTPRB) which promotes endothelial cadherin junction maintenance (Gong et al., 2015). In a search for promoter sequences of genes that regulate adherens junction integrity, VE-PTP was found to be a transcriptional target of HIF-2α. Endothelial specific HIF-2α deletion had worse outcomes in the acute lung injury model due to decreased barrier function. The authors hypothesized that hypoxia induced stabilization of EC barrier is mediated by HIF-2α. Interestingly, VE-PTP also dephosphorylates TIE2 and ANG2 and regulates VEGFR2. This suggests that chronic ischemia, by inducing HIF-2α, may protect against hypoxic lung injury.

#### Metabolic Regulation of the Cytoskeleton in Pulmonary Microvasculature Through Acetyl-CoA

The role of the cytoskeleton in acute lung injury involves acetylation of microtubules. Acetylation of α-tubulin is known to promote microtubule stability (Szyk et al., 2014). Acetylation requires the key intermediate metabolite acetyl-CoA, which is the sole donor of acetyl groups for acetylation (Choudhary et al., 2014). Acetylation of proteins involves the mitochondria, as acetyl-CoA, the substrate of acetylation, is generated either by pyruvate dehydrogenase and subsequent export of acetyl-CoA into the cytoplasm via the malate-aspartate transporter, or from glutamine reductive decarboxylation or through acetate, the two main mitochondrial-independent ways in which acetyl-CoA is generated (Pietrocola et al., 2015).

Acetylation also affects epigenetic functions of chromatin. Indeed, fatty acid-derived acetyl-CoA (which is generated in the mitochondria) was found to be critical for maintaining lymphatic differentiation *in vivo* (Xiong et al., 2018). Thus, mitochondria function is critical for acetylation. As mitochondria activity of ECs is enhanced by unidirectional flow and suppressed by disturbed flow (Wu et al., 2017), it is critical to determine how mitochondria function, acetylation, and lung injury are linked.

In acute lung injury models, multiple studies invoke a final common pathway of RhoA-induced activation of myosin light chain, causing EC contractions and pulmonary vascular leak. Microtubules (MTs) can modulate RhoA activity because Rho-specific guanine exchange factors (GEFs) can bind MTs; disruption of MT network by oxidative stress causes release of GEF-H1 which activates Rho signaling in an LPS-dependent manner (Kratzer et al., 2012). Furthermore, GEF-H1 bound to MTs is dependent on microtubule acetylation. For instance, cigarette smoke exacerbates LPS induced ALI *in vivo* and EC barrier dysfunction *in vitro* via increased susceptibility to histone deacetylase 6 (HDAC6) phosphorylation, which deacetylates MTs and reduces barrier function, thus promulgating ALI (Kratzer et al., 2012; Borgas et al., 2016; Karki et al., 2019). In concordance, HDAC6 null mice were resistant to LPS-induced ALI (Zhang et al., 2008). Selective HDAC6 inhibition by tubastatin A blocked TNFα-induced lung endothelial cell hyperpermeability, which was associated with increased α-tubulin acetylation and microtubule stability (Yu et al., 2016). Mechanically, increased stiffness also can activate GEF-H1 expression and thereby exacerbate LPS-induced lung inflammation (Mambetsariev et al., 2014).

Thus, acetylation levels, and hence, acetyl-CoA metabolism and mitochondrial function, may be critical for vascular permeability in ALI, and is downstream of mechanical loading. Interestingly, HDAC6 may also regulate the canonical Wnt/β-catenin pathway. β-Catenin HDAC6-dependent deacetylation causes β-catenin nuclear translocation and disassembly of adherens junctions (Li et al., 2008); on the contrary, β-catenin acetylation promotes its membrane localization thus stabilizing adherens junctions (Valenta et al., 2012; Iaconelli et al., 2015).

#### Endothelial Shear Stress in Pulmonary Hypertension

Shear stress in the pulmonary vasculature is altered in pulmonary arterial hypertension (PAH). Only through a combination of computational fluid dynamics and four-dimensional MRI has it recently become possible to measure wall shear stress in pulmonary arteries, as it was done to model carotid artery flow (Dai et al., 2004). PAH patients have more tortuous pulmonary artery branch vessels and larger proximal arteries (3.5 ± 0.4 cm compared to 2.7 ± 0.1 cm in healthy subjects) (Tang et al., 2012). This corresponds with radiology literature that increased main pulmonary artery diameter is highly specific for diagnosis of PAH (Kuriyama et al., 1984).

Additionally, the main pulmonary artery flow rate was significantly lower than in PAH patients by on average 1.1 L/m/m^2^. This combination of reduced flow rate and increased arterial diameter suggests that the shear stress is lower globally. Importantly, this may lead to a reduction in NO release from the endothelium (Hakim, 1994). In one patient during diastole, there was hardly flow at all (Tang et al., 2012). The calculated mean WSS of healthy subjects was almost 5-fold higher in healthy controls compared to PAH patients (20.5 ± 4.0 vs. 4.3 ± 2.8 dyne/cm^2^). Similarly, in distal pulmonary arteries, mean WSS was 1.4x higher in normal subjects than PAH subjects. Separate studies also found differences (2–3 fold) in shear stress between control and PAH patients (Barker et al., 2015; Odagiri et al., 2016; Schäfer et al., 2016; Zambrano et al., 2018). These values correlate with a reduction in NO bioavailability in PAH patients (Wolff et al., 2007; Gabrielli et al., 2011). This suggests that pruning of the distal vasculature in PAH may be a way for the lung to preserve microvascular perfusion—by increasing microvascular resistance, shear stress is elevated (Allen et al., 2014).

Counterintuitively, in PAH the microvasculature may also be subject to high shear states [such as in congenital heart disease (Gatzoulis et al., 2009)] or high oscillations in flow due to increased stiffness in the pulmonary arteries. Though the wall shear stress decreases in the larger pulmonary arteries, the pulsatility may increase in the microvasculature, which is how microvascular dysfunction is coupled to macrovascular dysfunction. Simply put, as the stiffness of arteries increases, cardiac pulsations are no longer dampened and instead are transmitted into the microvasculature. These high energy pulses can even be reflected back during, for instance, encounter with pulmonary thrombus (Castelain et al., 2001; Nakayama et al., 2001). Interestingly, in a rat model of PAH, reducing the flow to the lung via banding prevented development of plexiform lesions and inflammatory cell infiltrates, suggesting a causative role for force transmission in the development of PAH (Abe et al., 2016). However, the rats who had pulmonary artery banding had worse right ventricular function; moreover, hemodynamics were not measured. It is interesting that patients with a vasodilator challenge responsive PAH phenotype have increased survival (Rich and Brundage, 1987)—this suggests that reducing microvascular shear stress or pulsatility may improve PAH.

Mechanistically, microvascular cells isolated from patients with PAH demonstrated delayed shear adaptation and thus promoted shear induced endothelial injury and vascular remodeling (Szulcek et al., 2016). Additionally, pulmonary artery ECs subjected to high pulsatility but same mean shear stress exhibited increased NF-κB activation, inflammation, and extreme cell elongation, which could all be revered by microtubule stabilization with taxol (Li et al., 2009a, 2013). Decoupling the contributions from microvascular shear stress, pulsatility, oscillation index, and right ventricular function, not to mention cyclic stretch from breathing, would be necessary to fully work out the most important mechanistic and physiological parameters to survival in PAH.

#### ECs Are Metabolically Dysregulated in Pulmonary Hypertension

Morphologically, PAH is characterized by plexiform lesions, or disorganized endothelial cell growth, which is thought to be due to dysregulation of apoptosis and proliferation (Teichert-Kuliszewska et al., 2006; Abe et al., 2010). Indeed, isolated PAH patient cells have increased proliferation (Duong et al., 2011; Kim et al., 2013). Theoretically, the loss of BMPR2 (a hereditary mutation pre-disposing to PAH) pre-disposes cells to undergo apoptosis, and therefore causes the emergence of an apoptosis-resistant clonal cell population (Taraseviciene-Stewart et al., 2001). This suggests that a cycle of continual injury and healing happens in the pulmonary microvasculature is critical for the pathogenesis of PAH plexiform lesions. Correspondingly, patients with idiopathic pulmonary arterial hypertension (IPAH) showed increased uptake of fluorodeoxyglucose uptake in pulmonary arteries compared to controls. Furthermore, the glycolytic rate of endothelial cells derived from IPAH patients is 3-fold higher than controls. In IPAH cells, ATP content under hypoxia shows no decrease compared to normoxia, whereas ATP decreased by 35% in control (non-IPAH) cells, suggesting a greater dependence on respiration in control cells or reduced overall metabolic rate (Xu et al., 2007).

Mitochondria function in ECs is altered in PAH and can be reversed by promoting oxidative phosphorylation in rats (Rehman and Archer, 2010). Mechanisms include activation of HIF-1α, PDK-1, and downregulation of superoxide dismutase-2 (Rehman and Archer, 2010). The mitochondrial membrane potential, mitochondrial DNA, and regulators of mitochondrial biogenesis are reduced in a mouse BMPR2 ec^−/−^ PAH model (Diebold et al., 2015). Notably, BMPR2 knockdown in normoxia also pre-disposes ECs to increased glycolysis (Diebold et al., 2015). Glycolysis is increased by activation of pyruvate kinase muscle (PKM) via a miR-124 induced splicing switch (from PKM isoform 1 to 2). Downregulation of miR-124 resulted in increased expression of PTBP1 splicing factor and enhanced glycolysis (Caruso et al., 2017). How the downregulation of OXPHOS, yet upregulation of anaplerosis interact (as suggested, through YAP/TAZ below) is unknown.

HIF-1α also decreases the expression of Fe-S cluster assembly ISCU1/2 by induction of microRNA-210 and therefore the electron transport chain components. This causes reduced mitochondrial respiration (decreased aconitase and Complex I activity) (Chan et al., 2009). NFU1 and BOLA3 (Fe-S scaffold genes) are critical for Fe-S dependent synthesis of lipoic acid, an important fatty acid which is a covalent moiety of mitochondrial enzymes including the E2 subunit of PDH, which regulates oxidative metabolism, and BOLA3 expression is downregulated by hypoxia in ECs in PH (Cameron et al., 2011; Yu et al., 2019). BOLA3 controls Fe-S integrity and mitochondria complex protein levels and regulates respiratory complex activity in pulmonary artery ECs (PAECs). Lipoate modified PDH is critical for PDH function. It is unclear how lipoation and phosphorylation interact with each other for PDH activity. BOLA3 knockdown expectantly increased glycolysis but also increased oxygen consumption. BOLA3 knockdown increased PAEC proliferation and angiogenesis *in vitro* and *in vivo* assays (Yu et al., 2019).

#### NOTCH1 as a Mechanotransducer of Metabolism in Pulmonary Hypertension (PH)

Endothelial cell metabolism-epigenetic regulation of cell proliferation/angiogenesis (Hautefort et al., 2017; Xu et al., 2017) and migration (Zheng et al., 2012) is also regulated by cell-cell contact. NOTCH1 is a known sensor of high shear stress in arteries and stabilizes cellular junctions through contact dependence (Mack et al., 2017). NOTCH1 signaling is required to coordinate metabolism and gene regulation that is critical for proliferation of stalk cells in contact with migrating tip cells (De Bock et al., 2013a). NOTCH1 is also downstream of BMPR2 which is implicated in PAH. BMPR2 is required for NOTCH1 activation; deletion of NOTCH1 in ECs worsens hypoxia-PH (Miyagawa et al., 2019). Bmpr2^+/−^ mice demonstrate histological features of PAH such as impaired re-endothelialization, pulmonary neointimal thickening, and medial hypertrophy, suggestive of abnormal arterial remodeling. In endothelial specific NOTCH1 knockout mice, pulmonary hypertension was more pronounced under hypoxic conditions (Miyagawa et al., 2019).

It turns out that mutations or expression changes (Liu et al., 2017) in BMPR2 causes EC mitochondrial dysfunction (Diebold et al., 2015). NOTCH1 enhances glycolysis and mitochondria metabolism and promotes histone acetylation at enhancer binding sites for NOTCH1 and its target MYC, possibly responsible for wound healing after repeated injury (Miyagawa et al., 2019). Importantly, PFKFB3 is downstream of NOTCH1 signaling. NOTCH1 also increases mitochondrial replication and oxygen consumption rate due to a NOTCH1 intracellular domain-dependent mitochondrial transcription and replication. Interestingly, the authors postulated that PFKFB3 increased the production of acetyl-CoA through increased glucose-derived pyruvate flux through the mitochondria, which was also upregulated by NOTCH1 (Miyagawa et al., 2019).

### Stiffness Accounts for Morbidity and Mortality in Vascular Diseases

Endothelial substrate stiffness regulates cardinal microvascular functions including angiogenesis (Zhao et al., 2018), monolayer integrity (Birukova et al., 2013; Andresen Eguiluz et al., 2017; Urbano et al., 2017), immune cell transmigration (Stroka and Aranda-Espinoza, 2011; Schaefer and Hordijk, 2015). In the systemic arterial circulation, increased vascular stiffness is associated with and precedes systemic hypertension (Beltran et al., 2001; Weisbrod et al., 2013). Furthermore, in a computational model of baroreceptors as strain receptors, arterial stiffening is by itself sufficient to explain primary hypertension (Pettersen et al., 2014). An increase of macrovascular stiffness is an independent predictor of cardiovascular morbidity (Benetos et al., 2012; Smulyan et al., 2016) and mortality (Laurent et al., 2001; Pannier et al., 2005). Increased stiffness (or reduced compliance) which equates with systolic hypertension and lower diastolic blood pressure (Safar et al., 2013) also impacts the shear stress experienced by vascular endothelia by increasing the pulse wave velocity, as described above. Impaired endothelial function was independently and inversely related to pulse wave velocity, a measure of vascular stiffness in a large-scale study among healthy participants (Mceniery et al., 2006), and is probably a measure of endogenous nitric oxide response (Kinlay et al., 2001). Chronic vascular smooth muscle changes also contribute to vascular stiffness (Lacolley et al., 2017). Indeed, there is now an emerging concept of modulating stiffness properties of the vasculature in order treat hypertension. Current standard of care antihypertensive treatment not only improves mortality (Brunström and Carlberg, 2018) but is now thought to have an anti-stiffness component which modulates this important outcome (Chen et al., 2017).

Macrovascular stiffness promotes microvascular damage (Mitchell, 2008; Cardoso and Salles, 2016; Cooper et al., 2018) and therefore end-organ damage and eventual end-organ failure. The mechanism is likely dysregulated transmission of large shear stresses, pulsatility, or dysregulation of autoregulation of blood pressure at the capillary bed level. These large pulse waves contribute to EC permeability and inflammatory response to vasoactive agonists through stiffness-dependent control of RhoA GTPase activity (Birukova et al., 2013; Mambetsariev et al., 2014; Meng et al., 2015). Increased flow pulsatility also increases cytokine expression and enhanced leukocyte adhesion and reduced NO/upregulated endothelin expression in a microvascular model of stiffened pulmonary microcirculation (Li et al., 2009a,b). Stiffness also increases endothelial inflammation via NF-κB in an *in vitro* model of PAECs and isolated human tissue (Tan et al., 2014).

Pulmonary vascular stiffness plays an important role in the progression of pulmonary hypertension and is highly associated with mortality (Mahapatra et al., 2006; Gan et al., 2007; Campo et al., 2010; Thenappan et al., 2016), and when added to pulmonary vascular resistance is better at predicting clinical outcomes (Hunter et al., 2008). Wall stiffness increased from 0.5 to 2.0 MPa; correspondingly, the compliance decreased from 2.9 to 0.3 ml/mmHg (Zambrano et al., 2018), and capacitance and elasticity were also reduced in a PAH cohort (Schäfer et al., 2016).

#### Stiffness and Metabolism in ECs in Pulmonary Hypertension

Extracellular matrix stiffness increases EC glycolysis and reduces mitochondrial oxygen consumption with concomitant increases in glutaminolysis in cultured PAECs. Glutaminolysis is the conversion of glutamine to glutamate which feeds into the TCA cycle as α-ketoglutarate. These PAECs also exhibited increased proliferation and collagen deposition (Bertero et al., 2016). Glutaminolysis is essential for replenishing the TCA cycle during anaplerosis. Further evidence for glutaminolysis was found in PAH patients with hereditary BMPR2 mutation whom exhibited enhanced blood levels of glutamine, glutaminolysis and exhibited hyperproliferative phenotype in their ECs. Interestingly, the requirement for glutamine was driven by normoxic stabilization of HIF-1α and loss of SIRT3 in microvascular cells deficient of BMPR2 (Egnatchik et al., 2017) (Figures 1C,F).

Stiffness originates in the extracellular matrix. In a monocrotaline rat model of PH, and humans with PAH, there was increased collagen deposition (Bertero et al., 2015). Isolation of pulmonary arteries from PAH patients, and in hypoxia rodent models of PAH, revealed increased expression of markers of extracellular matrix remodeling via lysyl oxidases (Nave et al., 2014). Microvascular ECs, as well as smooth muscle cells, are noted to have increased alpha smooth muscle actin (SMA) expression, suggesting a role for a fibroblastic phenotype ]or endothelial-mesenchymal transition (endoMT)] in matrix deposition and contractility (Li et al., 2009b; Scott et al., 2013). Furthermore, targeting vascular matrix production with lysyl oxidase inhibitors can improve PAH (Nave et al., 2014). Stiffness is also directly linked to metabolic signaling through HIF-1α in the pulmonary microvasculature. Hypoxic metabolic modeling of ECs has revealed that they deposit more collagen under HIF-1α expression (Stenmark et al., 2006; De Jong et al., 2016). As recent literature has shown that collagen production requires enhanced glycolytic flux, as well as glutamine metabolism (Nigdelioglu et al., 2016; Hamanaka et al., 2019), stiffness induced metabolic changes undoubtedly plays a role in the pathophysiology of PAH.

#### Stiffness, YAP/TAZ, and Metabolism

Surface stiffness by itself can cause mesenchymal stem cell differentiation (Engler et al., 2006). For instance, neuronal differentiation programs are activated on soft surfaces (0.1–1 kPa), whereas muscle or bone differentiation programs are activated by hard surfaces (10–100 kPa). For reference, tissue culture plastic is in the ~10^6^ kPa range. The cytoskeleton plays a critical role in force transmission as these effects were mitigated by inhibitors of non-muscle-myosin II. Endothelial cells also demonstrate a strong cell shape dependence in their phenotype that is driven by surface stiffness and extracellular matrix contact (Chen et al., 1997). Investigations into factors which drive the transcriptional output of mammary epithelial cells based on low vs. high matrix stiffness led to the discovery of YAP and TAZ as being regulated by high stiffness (Dupont et al., 2011). YAP/TAZ have been shown to be activated in epithelial, fibroblast, endothelial, oncogenesis, neurons, and stem cells (Lian et al., 2010; Liu et al., 2015; Furukawa et al., 2017; Totaro et al., 2017; Chen et al., 2019). YAP/TAZ activity is regulated by interaction with the actin cytoskeleton—when perturbed by cytoskeleton remodeling, YAP is able to be de-phosphorylated and translocated into the nucleus. YAP/TAZ activate downstream pathways that are known to increase fibrotic pathways resulting in the synthesis of ECM (Totaro et al., 2018).

YAP/TAZ is controlled by and itself modulates cellular metabolism. In a rat pulmonary hypertension model, YAP promotes glutaminolysis through transcriptional activation of glutaminase (GLS1) in endothelia. Interestingly, in HUVECs glutamine was shown to be critical for cell growth but dispensable for cell migration (Kim et al., 2017). Stiffness-associated alterations in glycolysis through direct transactivation of lactate dehydrogenase are also in part due to YAP (Bertero et al., 2016). It has also been demonstrated recently that lipid accumulation is accelerated by YAP and TAZ in hepatocytes. Whether a similar mechanism exists in endothelial cells is unknown (Aylon et al., 2016; Jeong et al., 2018). Thus, it can be said that YAP/TAZ activation primes cellular metabolism for growth.

Cellular metabolism/energetics also drives YAP/TAZ: in the absence of favorable energy substrates or conditions, cellular growth or proliferation should be limited. Although the following studies were not performed in ECs, some of their findings may still be applicable, especially as it relates to energy/metabolic sensing. Depriving cells of glucose or inhibiting glycolysis reduces YAP/TAZ transcriptional activity through AMPK phosphorylation of AMOTL1, which inhibits YAP (Deran et al., 2014). AMPK also interacts with LATS in addition to directly phosphorylating YAP during energy starvation (Gailite et al., 2015; Mo et al., 2015). Conversely, energy abundance promotes YAP/TAZ transcriptional activity. PFK-1, the first enzyme that commits glucose to glycolysis, can bind YAP/TAZ TEADs to promote YAP/TAZ transcriptional programming (Enzo et al., 2015). In hyperglycemic conditions, the hexosamine biosynthesis pathway is induced and leads to O-GlcNAcylation of YAP, causing YAP-dependent transcription (Teo et al., 2010; Zhang et al., 2017). Unsaturated fatty acids may also stimulate β-catenin -dependent YAP/TAZ activity (Noto et al., 2017).

YAP/TAZ activation in epithelial and mesenchymal stem cells mimics increased ECM stiffness as in ECs. YAP is induced after injury and promotes wound healing (proliferation, migration) (Wang et al., 2012; Kimura et al., 2016) in smooth muscle atherosclerosis models *in vivo*. Mechanical feedback through matrix sensing/remodeling is responsible for smooth muscle growth in pulmonary hypertension models (Bertero et al., 2015; Kudryashova et al., 2016; Dieffenbach et al., 2017). YAP/TAZ also promotes the development of pro-fibrotic phenotypes in fibroblasts in *in vitro* models of idiopathic pulmonary fibrosis (Liu et al., 2015).

### Endothelial-Mesenchymal Transition (EndoMT) Is Related to Shear Stress, Stiffness, and Metabolic Changes

ECs display a significant amount of plasticity especially in pathological conditions. The endoMT, analogous to epithelial-mesenchymal transition, is a process by which endothelial cells have reduced expression of endothelial markers and increased expression of mesenchymal genes. EndoMT accounts for up to 30% of cancer associated fibroblasts in a murine melanoma model (Zeisberg et al., 2007a) and contributes to fibroblast stroma in a breast cancer model (Kidd et al., 2012). EndoMT is also thought to contribute to cardiac fibrosis (Zeisberg et al., 2007b). EndoMT has been shown to be involved in the pathogenesis of atherosclerosis and is thought to contribute to pulmonary fibrosis and pulmonary hypertension. Characteristic endoMT genes associated with fibroblast line include PDFGRα, SOX9, COL1A1, COL1A2, FSP1, vimentin, α-SMA, and periostin although the markers have different degrees of lineage specificity (Li et al., 2018).

EndoMT is dependent on mechanical forces including shear stress and stiffness. A time dependent shear stress/RNAseq study revealed that endoMT develops over time in cultured ECs exposed to oscillatory shear stress (disturbed flow) whereas pulsatile shear stress (unidirectional flow) did not induce endoMT genes. Metabolically, energy sensors AMPK and SIRT1 could inhibit oscillatory shear-induced endoMT. Mechanistically, compared to pulsatile flow, oscillatory flow increased DNA methylation of promoter regions of characteristic EC genes such as VWF, CD31, and Cadh5. However, there was decreased methylation of Cadh2, FSP1, and vimentin, markers of mesenchyme. The link between methylation and oscillatory flow is unknown (Lai et al., 2018). Correspondingly, statins and metformin are able to suppress endoMT (Osman et al., 2006).

EndoMT is also dependent on substrate stiffness. TGF-β-induced endoMT occurs preferentially on stiffer substrates and is inhibited by blockage of β-catenin/Wnt signaling pathway (Zhong et al., 2018). Furthermore, YAP is critical for developmental TGF-β-induced endoMT during heart valve mesenchyme formation (Zhang et al., 2014). Metabolically, TGF-β-induced endoMT is accompanied by inhibition of fatty acid oxidation, which is also required for endothelial cell proliferation through *de novo* nucleotide synthesis (Schoors et al., 2015). Inhibition of fatty acid oxidation alters intracellular acetyl-CoA levels, which maintains endothelial cell identity (Xiong et al., 2018).

#### Pulmonary Hypertension, Metabolism, and EndoMT

There is increasing evidence that endoMT contributes to EC dysfunction in the development in PAH and is regulated by HIFs (Good et al., 2015). Endothelial HIF-2α contributes to PAH through endoMT (Tang et al., 2018) leading to enhanced proliferation rate and down regulated prolyl hydroxylase 2 (and hence reduced ubiquitination-degradation of HIFs) (Dai et al., 2016). HIF-2α is thought to drive transcription of endoMT genes through SNAI1/2 (Snail) in a mouse hypoxia model whereas HIF-1α was dispensible; HIF-1α also did not regulate SNAI1/2 (Tang et al., 2018). However, in a separate rat hypoxia model, HIF-1α drove endoMT under Twist1 (Zhang et al., 2018a); additionally, SNAI1/2 was found to be a direct target of HIF-1α in endoMT of coronary endothelial cells (Xu et al., 2015). In an inducible HIF-1α endothelial-specific knock out mouse model, it was discovered that HIF-1α was necessary for formation of pulmonary hypertension in a chronic hypoxia model and fibrosis in a bleomycin model. Furthermore, HIF-1α expression was partially responsible for CTGF expression, an indicator of endoMT in PMVEC in pulmonary fibrosis induced pulmonary hypertension (Bryant et al., 2016).

Dysregulation of nitric oxide synthesis or reduction in NO bioavailability is also a hallmark of PAH. Nitric oxide generation requires arginine metabolism and is catalyzed by endothelial nitric oxide synthase. It turns out that in mice, HIF-1α regulates NOS2/iNOS and HIF-2α regulates Arg1 and Arg2. Arg2 is implicated in reducing airway NO and promotes remodeling and collagen deposition in PAH patients. Arg1 also decreases NO. Thus HIF-2α promotes PAH by upregulation of Arg1 and Arg2 (Cowburn et al., 2016). Interestingly, only the endothelial HIF-1α knockout model accrues severe right ventricular dysfunction (emblematic of PAH) in mouse models, whereas HIF-2α knockout does not; furthermore, patients with IPAH had higher HIF-2α levels than HIF-1α (Dai et al., 2016).

#### Pulmonary Fibrosis, Stiffness, and EndoMT

Bleomycin lung injury models are a classical mouse model of pulmonary fibrosis, in which the lung parenchyma becomes markedly stiff. Isolating ECs on day 7 and 21 of after treatment of bleomycin, one group found that ECs had increased expression of TGF-β, connective tissue growth factor and platelet-derived growth factor-C, compared with untreated lungs. Furthermore, those isolated ECs had decreased NO production, increased α-SMA, and collagen production compared to control (Kato et al., 2018). It is thus possible that lung ECs give rise to some portion of the mesenchymal population in pulmonary fibrosis in addition to the resident intrapulmonary mesenchymal cells, which constitute between 10 and 20% of the total cell population in the lung (Barron et al., 2016). In comparison, the total lung endothelial population constitutes around 30% of the total lung cells (Crapo et al., 1982). Using a pan-endothelial LacZ expression system, a bleomycin injury experiment showed coincident immunostaining of X-gal, COL1A1, and α-SMA, suggesting bleomycin-induced expression of mesenchymal lineage markers by lung endothelial cells. In this series of experiments, endoMT accounted for around 16% of bleomycin-induced fibroblasts. These results were recapitulated *in vitro* and established a role for ECs in promoting pulmonary fibrosis through endoMT in a Ras and TGF-β-dependent manner (Hashimoto et al., 2010).

The molecular mechanisms underpinning endoMT in pulmonary fibrosis are just starting to be elucidated but may be related to stiffness sensation. Endothelial HSP1 (HSPB1) suppresses markers of endoMT such as α-SMA after TGF-β stimulation. Furthermore, HSPB1 knockdown increased radiation induced endoMT in lung (Choi et al., 2016). A possible mechanism is through HSPB1 activation of Snail degradation (Wettstein et al., 2013). Snail is a transcription factor known to induce fibrosis in kidneys (Boutet et al., 2006) and epithelial-mesenchymal transition in pulmonary fibrosis (Jayachandran et al., 2009). HSPB1 also interacts with actin and is thought to regulate actin filament dynamics as it inhibits actin polymerization *in vitro*. HSPB1 colocalizes with cortical actin and is involved in EC adhesion and motility (Trott et al., 2009). Perhaps HSPB1 acts as a stress sensor, as cells depleted of HSPB1 are unable to close wounds or tissue planes (Doshi et al., 2009). This raises the possibility that disruption of tension homeostasis through the actin cytoskeleton might play a role in endoMT and may be a universal driver in development as well as fibrosis. Indeed, YAP/TAZ has been implicated in fibroblast activation during lung fibrosis (Liu et al., 2015; Noguchi et al., 2017) and thus likely plays a role in endoMT. On the flip side of protection against TGF-β-dependent endoMT, one study implicated TGF-β-dependent CXCR7 expression in ECs as a mechanism for negative feedback inhibition of TGF-β-induced endoMT by suppressing Notch signaling (Guan and Zhou, 2017).

## Conclusion and Emerging Hypotheses

Altered mechanosensation with a reduction in shear stress mimics a reduction in cardiac output or reduced oxygen delivery. Similarly, lower mass flux is a perfect set up for accumulation of infectious material. Thus, it is conceivable that endothelial cells reduce oxygen consumption and increase glycolysis via HIF-1α and activate inflammatory signaling. Concomitant reduction in acetyl-CoA through a reduction in mitochondrial metabolism transmits this energetic condition into a different epigenetic state in addition to loss of barrier protection. Increased substrate stiffness leads to activation of YAP/TAZ which promotes cell proliferation through coordination of glycolysis with anaplerotic pathways. This results in a stiffening of the local circulatory system which may increase local blood flow to pathological levels in the microvasculature. Changes in stiffness also lead to activation of endoMT which again changes the metabolism of the cell with an increase in matrix deposition, causing localized fibrosis.

Not only are biosynthetic and energetic pathways in endothelium modulated by mechanical forces, but metabolic modifications of proteins also change their function. For example, endothelial barrier disruption clearly involve changes in microtubule acetylation states. It is possible that metabolic signaling through protein modifications play an essential role in regulating cellular phenotype in stretch-dependent lung injury; however, this is an under-investigated topic.

With technological advancements such as single cell RNA sequencing, single cell proteomics, and metabolomics, and new tissue clearing methodologies, many novel metabolic hypotheses will finally be able to be tested in animal disease models at the cellular level. With the lung as a privileged organ as a first pass filter for many injectable intravenous medications as well as inhaled medications, targeting pulmonary microvascular niches may be possible.

This review focused on mechanotransduction in the lung vasculature and its effects on endothelial metabolism in pulmonary hypertension, pulmonary fibrosis, and acute lung injury. While endothelial dysfunction is also a feature of chronic obstructive lung disease and asthma, the role of metabolism and mechanical factors is less clear and warrants further study. Furthermore, shear stress, traction forces, and stretch (though not discussed in this review) are often studied independently; it is unclear how combined loading affects cellular metabolism.

## Author Contributions

DW conceived, wrote, and edited the manuscript. KB conceived and edited the manuscript.

### Conflict of Interest Statement

The authors declare that the research was conducted in the absence of any commercial or financial relationships that could be construed as a potential conflict of interest.
